# Strategies and sustainability in fast charging station deployment for electric vehicles

**DOI:** 10.1038/s41598-023-50825-7

**Published:** 2024-01-02

**Authors:** Abdallah Mohammed, Omar Saif, Maged Abo-Adma, Ashraf Fahmy, Rasha Elazab

**Affiliations:** Faculty of Engineering, Helwan University, Cairo, Egypt

**Keywords:** Electrical and electronic engineering, Engineering

## Abstract

This comprehensive review investigates the growing adoption of electric vehicles (EVs) as a practical solution for environmental concerns associated with fossil fuel usage in mobility. The increasing demand for EVs underscores the critical importance of establishing efficient, fast-charging infrastructure, especially from the standpoint of the electrical power grid. The review systematically examines the planning strategies and considerations for deploying electric vehicle fast charging stations. It emphasizes their unique dual role as loads and storage units, intricately linked to diverse road and user constraints. Furthermore, the review underscores the significant opportunity surrounding these stations for the integration of distributed renewable energy sources. It thoroughly explores the challenges and opportunities intrinsic to the planning and localization process, providing insights into the complexities associated with these multifaceted stations. Renewable resources, including wind and solar energy, are investigated for their potential in powering these charging stations, with a simultaneous exploration of energy storage systems to minimize environmental impact and boost sustainability. In addition to analyzing planning approaches, the review evaluates existing simulation models and optimization tools employed in designing and operating fast charging stations. The review consolidates key findings and offers recommendations to researchers and grid authorities, addressing critical research gaps arising from the escalating demand for electric vehicle fast-charging infrastructure. This synthesis is a valuable resource for advancing understanding and implementing robust strategies in integrating EVs with the electrical power grid.

## Introduction

In the current global scenario, an urgent imperative exists to address escalating environmental concerns, leading to an intensified quest for sustainable solutions to mitigate the adverse impacts of human activities. This imperative is substantiated by an expanding body of literature^1,2^. Within this overarching context, the transportation sector has emerged as an obvious contributor to greenhouse gas (GHG) emissions, primarily due to its dependence on fossil fuels. This reliance not only poses a formidable challenge to environmental sustainability but also perpetuates global oil consumption trends that are ecologically and economically untenable^1^.

In response to these substantial challenges, electric and hybrid vehicles (EVs) have garnered prominence as viable alternatives in contemporary transportation. These vehicles leverage clean energy sources, exhibiting environmentally friendly characteristics that play a pivotal role in reducing pollution levels and curbing the carbon footprint associated with the transportation sector^3^. Despite encountering transient disruptions from the COVID-19 pandemic, the collective progress achieved by the EV market, as evidenced by battery electric vehicle (BEV) and plug-in hybrid electric vehicle (PHEV) sales surpassing two million units in 2019^3^, instills optimism for sustained growth in the next decade. A more granular analysis of BEV volumes reveals a 74% share of global EV sales in 2019, marking a 6%-point increase compared to the previous year. This growth surge can be attributed, in part, to the implementation of stringent European emissions standards, catalyzing manufacturers to prioritize zero-emission vehicles. This paradigm shift underscores the pivotal role of EVs in the broader context of environmental sustainability^4^.

The burgeoning global significance of EVs is palpable, especially in the context of smart city development, where they serve as a linchpin in establishing sustainable and energy-efficient urban environments. EVs’ continued popularity and adoption are poised to exert a profound and positive influence by limiting carbon gas emissions and enhancing urban air quality^4^. However, several formidable challenges persist despite the myriad benefits offered by EVs. A prominent obstacle relates to insufficient infrastructure for charging, a fundamental hindrance to achieving widespread EV implementation. The availability and accessibility of charging stations are pivotal to facilitating convenient and efficient charging for EV owners, necessitating the development of a robust and easily accessible public charging infrastructure.

Another critical challenge linked to the widespread implementation of EVs is the potential strain on the existing utility grid. Charging EVs on a large scale demands a substantial and consistent power supply from the grid, with the potential to overload the grid distribution system. Additionally, the relatively longer charging times for EVs than traditional refueling necessitates the development of ultra-fast charging stations capable of delivering charging speeds comparable to the conventional refueling process^5,6^.

Despite these challenges, the growing global emphasis on environmental issues has sparked heightened interest in using clean and renewable energy. EVs have emerged as a fitting solution to mitigate emissions from the transportation sector, attracting significant attention from both the academic and industrial sectors. EVs exhibit superior energy efficiency, environmental friendliness, and cleanliness compared to traditional fueled vehicles, particularly when integrated with smart grids.

The adoption of electric vehicles has the potential to substantially minimize the environmental and economic costs associated with traditional fueled vehicles, thereby contributing to a more sustainable future^7^. The planning of EV charging stations encompasses a multifaceted set of objectives addressing grid electrical technical considerations, station owner's economic goals, driver-centric requirements, and overarching environmental imperatives.

**Figure 1:**
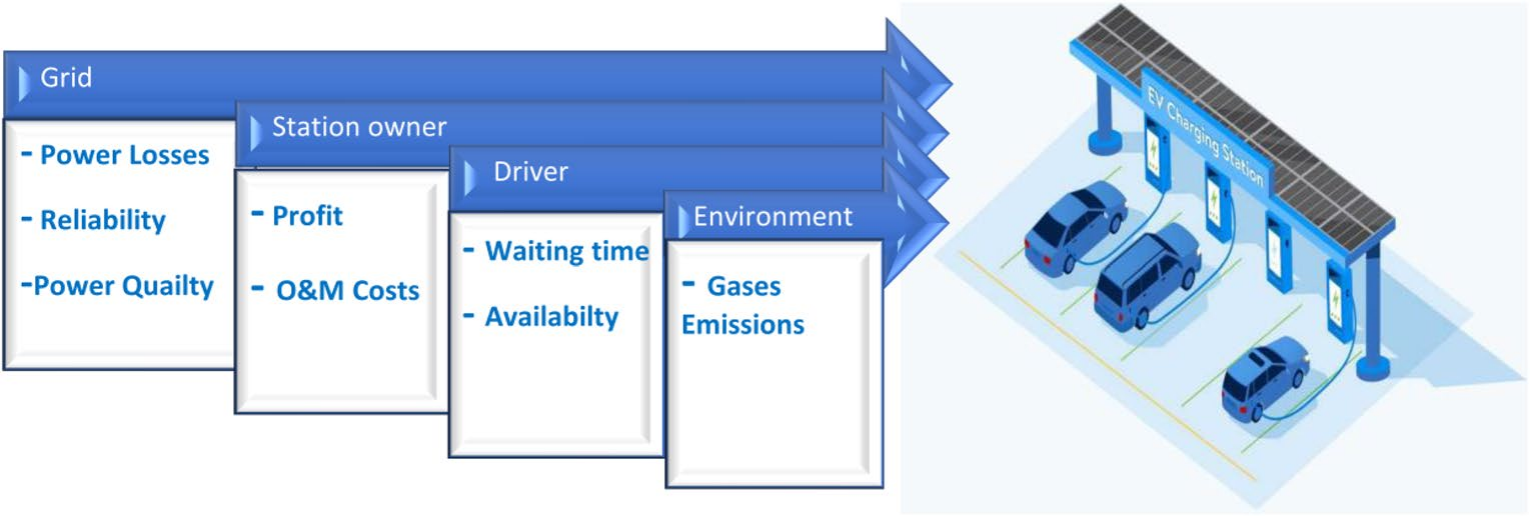
EV planning considerations.

From a grid perspective, the planning process focuses on ensuring electrical stability, minimizing power losses, optimizing grid reliability, and maintaining power quality. These technical objectives are vital to ensuring the seamless relationship of EV charging infrastructure with the existing electrical grid. Simultaneously, station owners seek economic viability and profitability, maximizing revenue while minimizing operation and maintenance (O&M) costs. Their goals encompass efficient station utilization, revenue generation, and business sustainability. For drivers, the planning of EV stations must prioritize convenience, reduced waiting times, station availability, and efficient charging modalities aligning with their daily routines. Figure 1 summarizes different recent planning considerations.

In addition to these considerations, environmental objectives play a pivotal role, compelling the incorporation of renewable energy resources and energy-efficient technologies into charging stations. This dual focus on lowering CO_2_ emissions and minimizing the environmental footprint of EV charging infrastructure underscores the collective responsibility to address climate change and contribute to a cleaner, more sustainable future. Thus, the planning of electric vehicle stations is a harmonization of these diverse objectives, seeking to create a balanced and holistic solution that serves the interests of all stakeholders while advancing environmental sustainability.

Charging stations can be approached from various perspectives, with numerous studies focusing on optimizing the charging/discharging processes to improve the integration of EV charging stations, as demonstrated in Refs.^8–12^. In Ref.^13^, the transportation electrification market is also studied. On the other hand, from a power system standpoint, the planning of EV charging stations presents unique characteristics, wherein these stations function both as loads and storage units, further entwined with various road and user constraints. This study primarily delves into the power system planning aspect, exploring recent advancements in literature.

**Figure 2:**
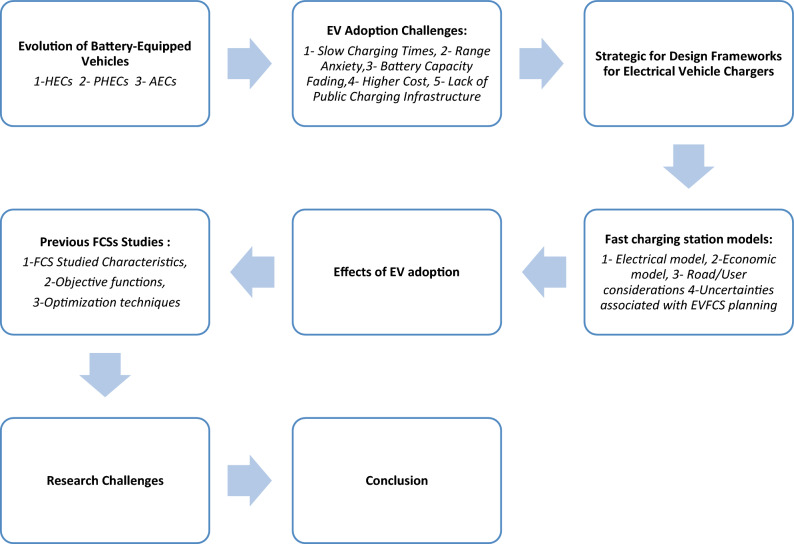
The study framework.

The paper’s structural framework involves an examination of EV configurational variations in “Evolution of battery-equipped vehicles” section, addressing challenges linked to EV adoption in “EV adoption challenges” section, providing a concise overview of the key characteristics of various types of charging stations in “Strategic for design frameworks for electrical vehicle chargers” section, exploring technical and economic models about fast charging stations in “Fast charging station models” section, summarizing the EV adoption effects in “Effects of EV adoption^53^” section, comparing recently proposed charging station planning studies in “Previous FCSs studies” section, and finally, highlighting the main research challenges and conclusions investigated by this work in “Research challenges” section, as shown in Fig. 2.

## Evolution of battery-equipped vehicles

Over the past decade, a diverse array of battery-equipped vehicles has surfaced, categorically falling into distinct classes such as all-electric vehicles (AECs), hybrid electric vehicles (HECs), and plug-in hybrid electric vehicles (PHECs). Additionally, there is a niche category of EVs powered by fuel cells, promising lower emissions and heightened efficiency, albeit hindered by challenges like the high cost of hydrogen production, infrastructure requirements, and limited commercial availability^14^.

**Figure 3:**
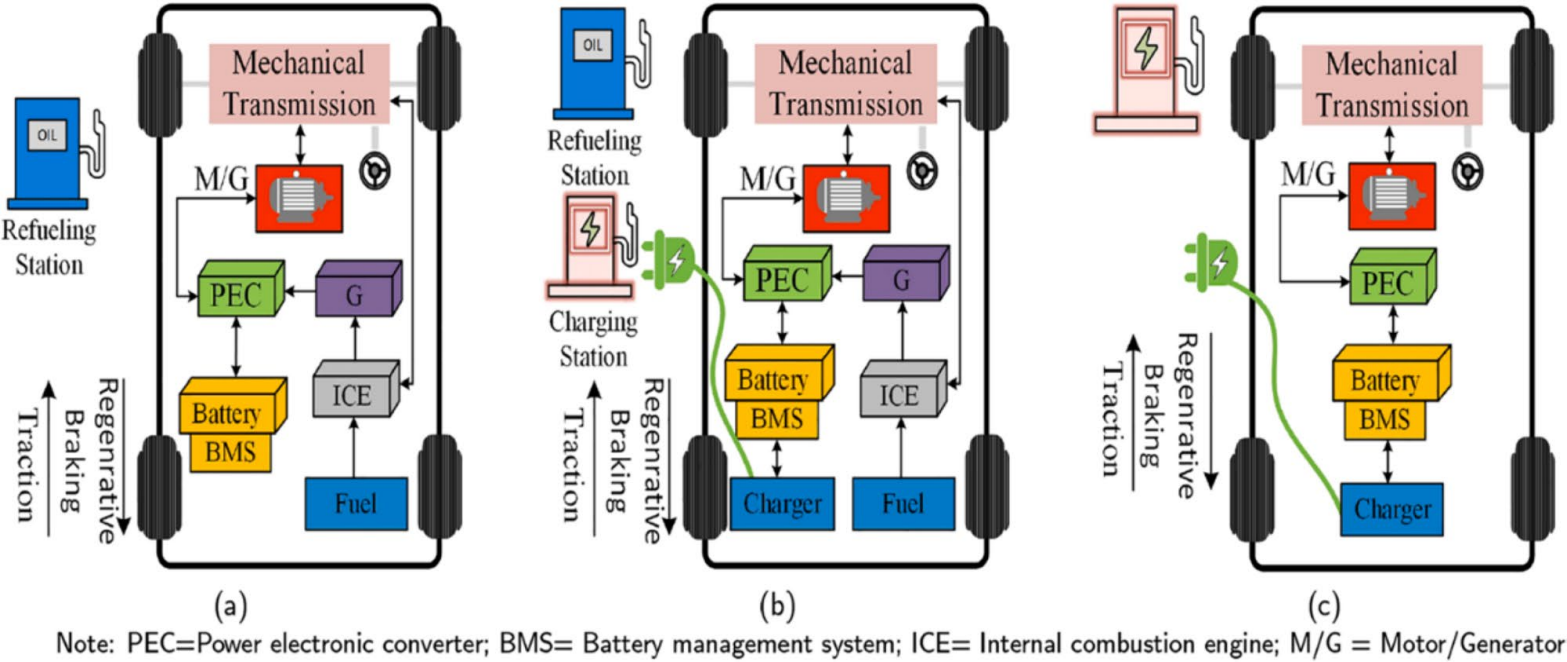
Power drive of EV (**a**) EV hybrid, (**b**) EV Hybrid Plug-in, (**c**) All-EV^15^.

Toyota, Honda, Ford, Mitsubishi, BMW, Nissan, and Volkswagen are among the manufacturers. It primarily focuses on expanding its HECs and PHECs lineup, while Tesla emphasizes AEC models more. The main architectural characteristics of these three primary types of EVs are illustrated in Fig. 3.

### Hybrid electric vehicles (HECs)

Among the prevailing battery-equipped vehicles, hybrid electric cars (HECs) have emerged as the predominant type globally, representing a commendable stride towards curbing gas emissions, particularly in urban environments. HECs exhibit a unique architecture featuring internal combustion engines (ICE) utilizing fossil fuels and battery packaging. The battery pack, illustrated in Fig. 3a, is charged utilizing the ICE rather than relying on an external charging source^16^.

HECs incorporate an electric generator alongside a high-power battery pack to bolster energy efficiency, capturing kinetic energy during braking. Traditionally lost as heat in conventional braking systems, this kinetic energy is redirected to the battery pack in HECs, a particularly advantageous feature in densely populated urban areas with high traffic, where frequent braking occurs^17^.

### Plug-in hybrid electric vehicles (PHECs)

As depicted in Fig. 3b, a Plug-in Hybrid Electric Car (PHEC) seamlessly blends a fossil fuel-powered engine with a battery package. The battery pack of a PHEC can be externally charged through a grid connection or an off-grid charger. Operating in two distinct modes—all-electric and hybrid—the PHEC utilizes the battery package as the primary power source for shorter distances in all-electric mode. Conversely, when the battery package’s state of charge (SoC) falls below a specified level in hybrid mode, the PHEC seamlessly transitions to the fossil fuel-based engine, ideal for longer routes where the internal combustion engine offers requisite power^18^. Consequently, PHECs deliver enhanced fuel economy compared to conventional fossil fuel-based vehicles^19^.

Like HECs, PHECs are engineered with a regenerative braking system to capture and store kinetic energy generated during braking. This system allows the PHEC to convert and store energy that would typically be dissipated as heat, thus recharging the battery pack and augmenting overall energy efficiency.

### All-electric cars (AECs)

Illustrated in Fig. 3c, all-electric vehicles (AECs) represent a paradigm shift in automotive technology, relying exclusively on battery packs as the primary energy source and propelled by electric motors. AECs offer distinct advantages over conventional Internal Combustion Engine (ICE) cars HECs and PHECs. These advantages encompass seamless operation, heightened efficiency, absence of noise pollution, and the lowest local GHG emissions.

Recognized for their exceptional efficiency, typically ranging from 60 to 70%, AECs outperform ICE-based cars significantly^20^. Key characteristics of different EVs are summarized in Table 1, providing a comprehensive overview of their technical specifications.

**Table 1:** Characteristics of EV types.

Features	Hybrid EV	Plug-in Hybrid EV	All EV
Sources of energy	Petroleum derivatives Battery package	Petroleum derivatives Battery package	Battery package Capacitor
System of propulsion	Internal combustion engine drive Electric drive	Internal combustion engine drive Electric drive	Electric drive
An external source of energy	Petroleum derivatives Fuel station	Petroleum derivatives Service Station Charging Station	Charging Station
Characteristics	More fuel-efficient than conventional cars Various energy sources Low emissions Long distance range Regenerative braking^21^ Battery voltage (12.0, 48.0–160.0, 0.0–300.0 V)^15^	Various energy resources Less fuel usage and low emissions Regenerative braking Battery voltage (300.0–400.0 V)^15^ Range (16.0–80.0 km)^22^	Low CO2 emissions Short distance range Depend only on batteries Regenerative braking Battery voltage (Tesla Roadster (375.0 V)^23^, Nissan Leaf (360.0 V) ^23,24^) Range (100.0–640.0 km)
Major concerns	Cost of petroleum derivatives Gas emissions Management of nonrenewable energy sources Optimization of engine and battery size	CO2 emissions Cost of petroleum derivatives Batteries costs	Battery packages cost Range Limited public charging services Expensive Time for charging
Capacity (kWh)	Toyota prius (1.30)^23^ Toyota camry hybrid (1.60)^23^ Ford fusion hybrid (1.40)^23^	Mitsubishi outlander PHEV (12.0) ^25^ Chevrolet volt (17.10)^26^ Toyota prius prime (8.80)^26^	Nissan LEAF (40.0)^26^ Nissan LEAF e + (62.0)^26^ Tesla model S (85.0)^25^

## EV adoption challenges

The penetration of EVs in the vehicle market has been increasing gradually, albeit at a slower rate compared to the total vehicle population worldwide. Several challenges have hindered the increasing use of electric vehicles, including range anxiety, slow charging times, higher Vehicle costs, a shortage of infrastructure for charging, and battery degradation.

### Slow charging times

Unlike internal combustion engine (ICE) vehicles that can refuel in a few minutes, charging EVs takes longer. It varies based on battery capacity, vehicle type, and charging infrastructure. Residential charging typically takes around 7 h, while charging at dedicated charging stations can vary significantly, as discussed in “Strategic for design frameworks for electrical vehicle chargers” section.

### Range anxiety

Range anxiety refers to the worry of EV drivers that their battery will be depleted before arrival at their destination or the closest charging station^27^. Addressing range anxiety involves improving battery technology and the spread of public charging and battery swap stations. Integration of charging infrastructure with the network, providing precise vehicle performance details, and charging stations. The availability to help alleviate range anxiety^28,29^. Strategies such as enforcing charging time limits and ensuring sufficient charging capacity can also manage potential conflicts among drivers at public charging stations^30–35^.

### Battery capacity fading

The battery capacity in EVs degrades with each cycle of charging and discharging, eventually mandating replacement. Lithium-ion battery modules with multiple cells connected in parallel and series are commonly used in EVs. Effective battery management systems, regular maintenance practices, cell energy balancing, and SoC balancing are essential to optimize battery life and minimize the need for replacement^36–39^.

### Higher cost

EVs, particularly those equipped with lithium-ion batteries, have experienced a decline in cost over time. However, the initial cost of EVs, especially those with larger battery capacities, remains higher than ICE vehicles. The decreasing cost per kilowatt-hour (kWh) of batteries is a positive trend for the growth of the EV market^40^.

### Lack of public charging infrastructure

Inadequate charging station infrastructure is a significant barrier to plug-in EV market penetration. The infrastructure of public charging stations is critical in decreasing range anxiety and increasing consumer confidence. The value of public charging station infrastructure can be quantified to inform investment decisions and anticipate its impact on future EV sales.

## Strategic for design frameworks for electrical vehicle chargers

Charging stations are classified into various levels, where Slow charging, semi-Fast charging, fast charging, and ultra-fast charging are all available. Level I chargers are typically used at residential buildings, while Level II, Level III, and Level IV chargers exist in private and public areas, with varying charging speeds and capabilities. Table 2 illustrates the various charging levels and their applications, as discussed in references^41–45^.

**Table 2:** Charging levels of EV according to IEC 61 851-1.

Type of charging	Voltage (V)	Outlet place	Maximum power (kW)	Charging time (h)
Level I (slow charging)	120.0 US (AC)	Home	1.90	4.0–11.0
	230.0 EU (AC)		7.40	11.0–36.0
Level II (semi-fast charging)	240.0 US (AC)	Private/public	19.20	2.0–6.0
	400.0 EU (AC)		43.0	2.0–3.0
Level III (fast charging)	208.0–600.0 (DC)	Public	50.0–350.0	0.160–0.50
Level IV (ultra-fast charging)	≥ 800.0 (DC)	Public	> 400.0	Gas refueling

Table 2 illustrates that ultra-fast charging stations (FCS) employ high DC voltage and current to enable faster charging and simultaneously accommodate a larger number of vehicles. This technological advancement positions FCS as a promising trend for the future of charging infrastructure worldwide, with expected widespread implementation and rapid adoption. By utilizing high DC voltage and current, FCS reduces charging times and enhances the capacity of charging stations to serve a greater volume of vehicles concurrently. This transformative approach to charging infrastructure has attracted great interest and investment because of its potential to shape the future of EV adoption and facilitate seamless integration.

However, compared to slow overnight charging, FCS has distinct characteristics, including high charging power, centralized load demand, predominantly daytime charging, and a more pulsating load due to fast charging and higher power consumption. These specific features of FCS can create significant challenges in power quality, like voltage fluctuations, unstable harmonics, and increased harmonic emissions, as referenced in Refs.^46–50^. These issues should be addressed to allow reliable and powerful FCS operation and maintain grid power quality. The various charging station configurations are classified depending on electricity utilization. The following paragraphs discuss the features of these configurations.

### Battery swapping technology

Replacing the fully discharged or almost depleted batteries in fully charged electric vehicles is called battery swapping. As electric vehicles become increasingly widespread, establishing battery swap stations becomes critical. In Ref.^50^, researchers have developed optimal battery swap station models within distribution systems. A modified differential evolution algorithm is used to solve the proposed method. Various optimization strategies were proposed to achieve the most efficient operation of swapping stations for battery packs, in Refs.^51,52^.

### It is a charging station that operates solely on grid electricity

**Figure 4:**
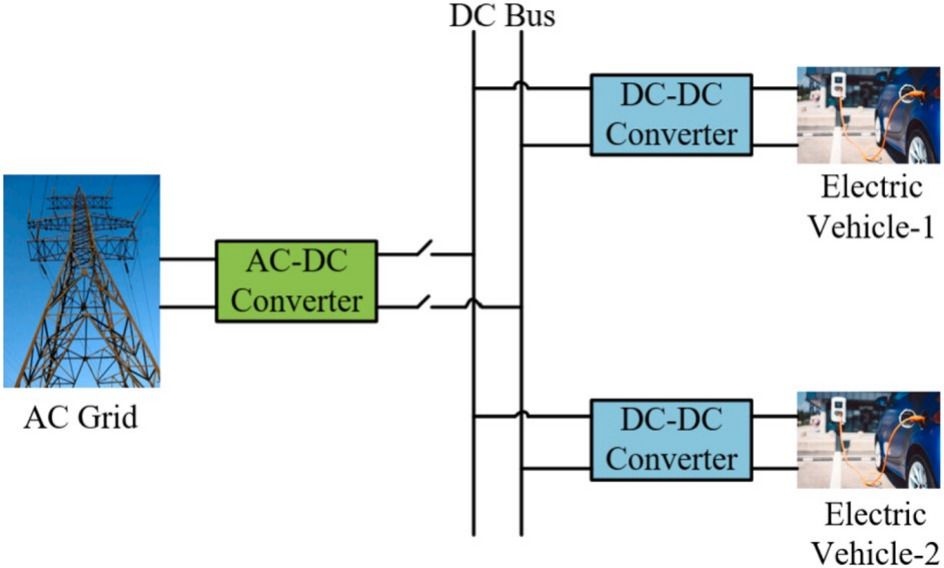
Charging station that operates solely on grid electricity.

As the electric vehicle market experiences rapid growth, there is an imperative need to establish fast DC charging stations. These stations are comparable to traditional petroleum refueling stations, enabling electric vehicle charging within minutes, making them the fastest charging option. Given the surge in demand for electricity the grid supplies, meticulous planning in designing these charging stations is crucial. Figure 4 provides an overview of a charging station powered exclusively by the grid^32^.

### They are charging stations utilizing an energy storage system and grid electricity

**Figure 5:**
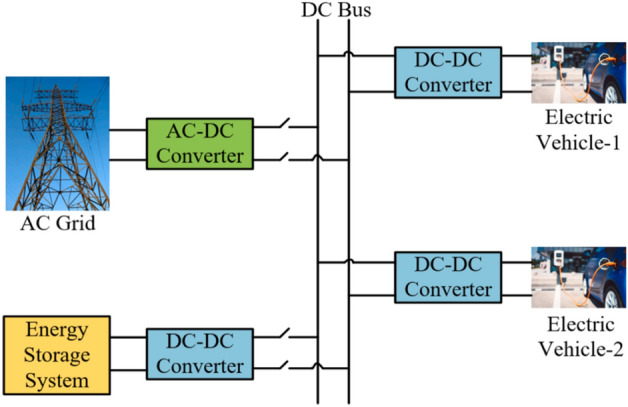
Charging station utilizing grid power and energy storage system [ESS].

The distribution network faces an enormous issue because of the rising demand for electrical power at charging stations. Consequently, the requirement for electrical energy has increased, resulting in the adoption of Energy Storage Systems (ESS)^53^. Figure 5 illustrates a charging station with grid power and an energy storage system. ESS cannot only enhance the distribution network’s effectiveness but also impact the station’s cost-effectiveness. As a step toward implementation, ESS has been integrated into fast-charging stations as a prototype^54^. Numerous studies have been conducted to increase the cost-efficiency of energy storage systems and fast charging stations^55–58^.

### Charging stations utilize both grid and renewable energy

**Figure 6:**
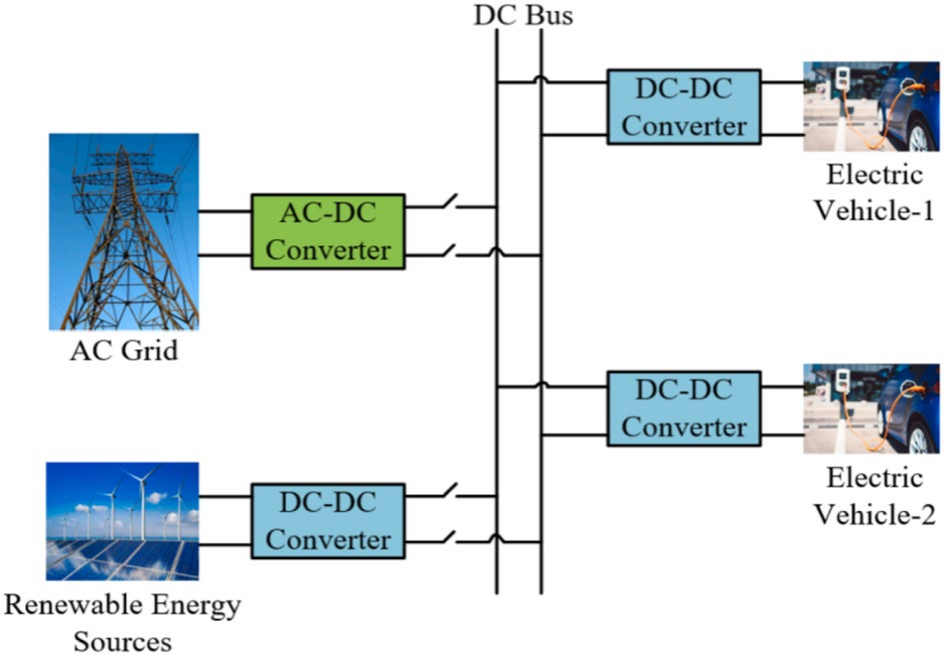
Charging station utilizing grid power and renewable energy.

Electricity demand has increased due to the rapid growth of electric vehicles. To address this growing energy requirement, charging stations that harness both power grid and renewable energy sources (RES) are being developed. Figure 6 illustrates a charging station that combines grid power with renewable energy sources. The goal is to achieve an optimal and reliable power exchange. In this context, fast EV charging stations have been seamlessly integrated with the grid^59^. A solar energy production plant with a station for fast charging is needed to implement a successful energy management strategy.

### Integrated station: grid power, RES, and ESS

**Figure 7:**
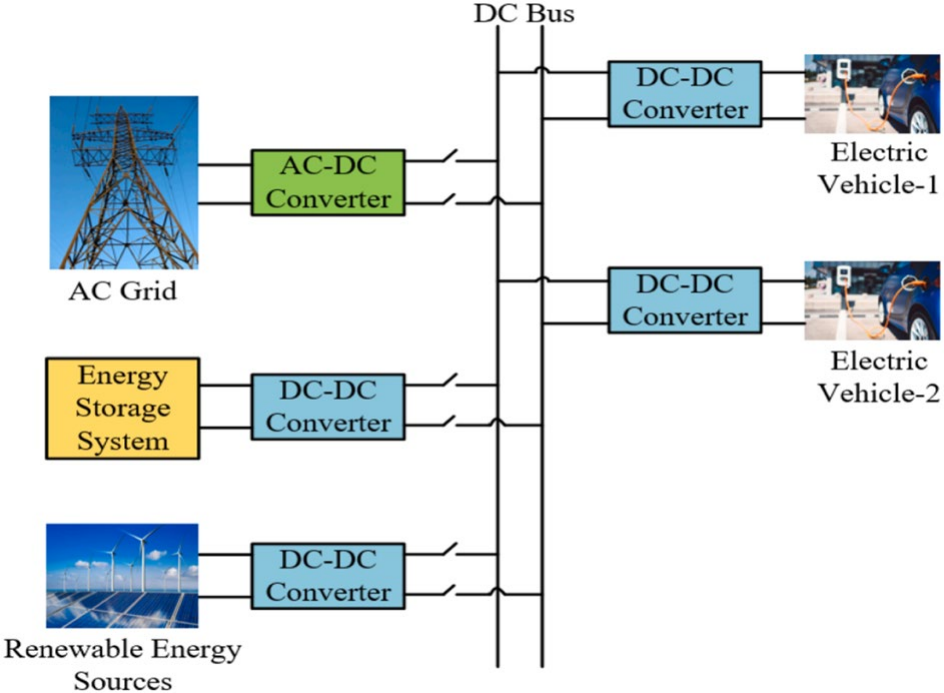
Charging station combines grid power, RES, and ESS.

The increasing demand for electricity at charging stations has the potential to influence grid performance considerably. As a result, it becomes imperative to integrate RES into charging stations to bolster the grid’s capacity, especially during peak demand periods. ESS is incorporated into these charging stations to solve the problems with the electrical grid. ESS installations serve to store and release energy, effectively mitigating grid-related issues. Furthermore, integrating ESS into charging stations reduces the unpredictability of renewable energy generation, with batteries being the primary storage medium. Figure 7 illustrates a charging station that combines renewable energy, grid electricity, and an energy storage system. Numerous studies have been published to investigate this topic further^60–62^.

### Charging station off the grid

Instead of using the conventional utility grid, distributed energy sources provide power for off-grid charging stations. Consequently, developing and placing off-grid charging stations into operation requires meticulous attention. In Ref.^63^, an optimal algorithm is introduced for designing and implementing off-grid charging stations catering to electric and fuel cell vehicles. This algorithm addresses uncertainties in parameters and station design through stochastic programming, and it includes integrating a diesel generator to reduce costs by 15%. Another approach, presented in Ref.^64^, suggests implementing unconventional energy sources in an electric vehicle charging station disconnected from the grid (EVCS) for a village. This strategy harnesses wind and solar energy and an Energy Storage System (ESS) to eliminate the need for diesel generators. However, there are various challenges when proposing a charging station that relies on renewable energy sources. Lastly, Ref.^65^ presents a charging station for plug-in hybrid electric vehicles that blends renewable energy sources with a fuel cell system.

## Fast charging station models

Different models have already been formulated to discuss the characteristics and the impact of electric vehicle charging, particularly about FCS. The specific characteristics and objectives of each study vary. In Ref.^66^, a demand for a spatial–temporal charging model for EVs is formulated, while FCS locations on a circular freeway are determined using a nearest-neighbor clustering approach. However, The FCS placement does not consider the power system limits.

Another study^67^ introduced a stochastic method to consider the optimal size and locations for parking charging points in the grid distribution system. They employed the point estimate method for this purpose. A multi-stage FCS placement strategy is proposed in Ref.^67^, considering the increasing penetration rates of EVs and the interaction among the transportation sector, power sector, and charging station infrastructure. However, the service radius impact on FCS placement was neglected in the study.

Monte Carlo non-sequential algorithm is mentioned as a method to build examples in the transportation network and model the development of traffic flow under the circumstances. This aids in determining the spatial–temporal distribution of the load for EV charging in various locations^68^. The travel chain theory may also determine the temporal and geographic distribution of demand for EV charging in various locations and dates^69^.

It is worth noting that the literature often treats the FCS load needed as a Constant Power Load (CPL) when investigating its effect on the electrical distribution systems. However, one study^70^ suggests using an exponential load model better to present the charging demand in a power flow study. Various environmental, social, and economic factors can be incorporated into the models to enhance the planning behavior of FCS, as discussed in the subsequent subsections.

Regarding the electrical model, daily load curves are estimated for different charging stations to illustrate the effect of EV charging power on the distribution grid. The power balance equation used in the power flow analysis needs to be adjusted to account for the voltage-dependent nature of the fast-charging station load, as described below^71^.

1 $${P}_{Gi,t}={P}_{D0i,t}+{\varphi }^{k}E\left[{P}_{{FCS}_{t}^{k}}\right]\left[{a}_{p}{\left(\frac{{V}_{i,t}}{{V}_{o}}\right)}^{{n}_{p}}+{b}_{p}\right]+{V}_{i,t}\sum_{j=1}^{{N}_{B}}{V}_{j,t}\left({G}_{ij}{\text{cos}}\left({\theta }_{ij,t}\right)+{B}_{ij}{\text{sin}}\left({\theta }_{ij,t}\right)\right), $$

where ${{\text{V}}}_{{\text{i}},{\text{t}}}$; ${{\text{V}}}_{{\text{j}},{\text{t}}}$: voltage magnitude for bus i and j, ${\uptheta }_{{\text{ij}},{\text{t}}}$: phase angle difference between bus i and j, ${{\text{P}}}_{{\text{Gi}},{\text{t}}}$: active power generated for a generator at bus I, ${{\text{P}}}_{{\text{D}}0{\text{i}},{\text{t}}}$: active load required at bus i and k is one if FCS is located at bus i of distribution system and 0 otherwise.

The average charging power for individual vehicles is calculated to estimate the total station demand power-time profile. The total charging power required for *No* number of electric vehicles being charged can be calculated as follows^72,73^:

2 $${P}_{{FCS}_{k,t}}=\sum_{j=1}^{{N}_{o}}avg{p}_{{SOC}_{ini} }^{j},$$

where ${{\text{avgp}}}_{{{\text{SOC}}}_{{\text{ini}}}}^{{\text{j}}}$: average required charging power, ${{\text{P}}}_{{{\text{FCS}}}_{{\text{k}},{\text{t}}}}$: the total required charging power for No number of EVs being charged at kth FCS at time t.

So, the total expected electric vehicle charging load required at time t for all possible values of *No* (where *No* from 1 to *Nc*) is given by:

3 $$E\left[{P}_{{FCS}_{t}^{k}}\right]=\sum_{{N}_{o}}^{{N}_{c}}{P}_{t}^{k}\left({N}_{o}\right)\times {P}_{{FCS}_{t}^{k}},$$

where ${{\text{P}}}_{{\text{t}}}^{{\text{k}}}\left({{\text{N}}}_{{\text{o}}}\right)$: The probability of the number of EVs being charged at kth FCS in time t.

### Economic model

The overall social cost of charging infrastructure includes both economic and environmental factors. Economic expenses can be broken down into two components: the capital cost [F1] and the charging costs [F2]. The charges for building and maintaining the charging stations are included in the capital cost. The size of the stations, which is specified by the number of chargers, plays a significant role in determining the building cost. The building cost can be calculated using the following formula^74^:

$$ {\text{Capital cost }}\left( {{\text{F1}}} \right) \, = {\text{ Fixed investments }} + {\text{ Ongoing operational expenses}}{.} $$

The fixed investments include the initial capital expenditure required to set up the charging infrastructure, such as land cost, construction, electrical infrastructure, and charging equipment. These costs are incurred upfront and are considered one-time investments.

The ongoing operational expenses include the recurring expenses related to the maintenance, operation, and management of the charging stations. This includes electricity consumption, maintenance, repairs, customer support, billing systems, and any other operational costs.

By considering both the fixed investments and ongoing operational expenses, the building cost can be estimated, providing an understanding of the economic component of the overall social cost of EV charging stations. Building cost can be presented by a mathematical formula as follows^74^:

4 $${F}_{1}={\sum }_{j\in J}{C}_{j}\left[T\left({N}_{j}\right)\frac{{{r}_{o}\left(1+{r}_{o}\right)}^{{n}_{year}}}{{\left(1+{r}_{o}\right)}^{{n}_{year}}-1}+Y\left({N}_{j}\right)\right],$$

where ${{\text{C}}}_{{\text{j}}}$: indicates if a charging station is at the position of j or not, ${{\text{N}}}_{{\text{j}}}$: is the number of chargers to be built in site j, ${\text{T}}\left({{\text{N}}}_{{\text{j}}}\right)$: is the capital function of fixed cost, ${\text{Y}}\left({{\text{N}}}_{{\text{j}}}\right)$: is the annual operating cost of site j, ${{\text{r}}}_{{\text{o}}}$: the discount rate, year: the depreciation period of charging stations.

The charging costs include two components: the transportation expenses to reach the charging stations and the electricity costs. The transportation expenses encompass the power consumption and time required to travel to the charging station, while the electricity expenses refer to the fees incurred by individuals when utilizing the chargers. Charging costs can be mathematically formulated as follows^74^:

5 $${F}_{2}=365[\omega {\sum }_{j\epsilon J}{\sum }_{i\epsilon I}{X}_{ij}{n}_{i}{d}_{ij}\sigma +k{\sum }_{i\epsilon I}{n}_{i},$$

where ${{\text{X}}}_{{\text{ij}}}$: indicates vehicle I choose to go to charging station j for charging service, $\upomega$: the power consumption per the unit distance to charging stations ${,\mathrm{ n}}_{{\text{i}}}$: the number of cars requiring charging every day at the point I, $\upsigma$: non-linear coefficient about roads, ${{\text{d}}}_{{\text{ij}}}$: the space linear distance between the point I to site j, ${\text{k}}$: the average electricity cost of a electric car currently.

The environmental cost associated with a charging station relates to the negative environmental impacts that it imposes. This includes factors such as greenhouse gas emissions, pollution, and the depletion of conventional resources resulting from generating and transmitting electricity used for charging. Additionally, the manufacturing and disposal processes of charging infrastructure and its components can contribute to environmental costs through activities such as raw material extraction, energy consumption, and waste generation.

To assess and quantify the environmental cost of a charging station, various factors need to be considered, including the electricity generation emissions, the type of energy source used, and the efficiency of the charging stations. Additionally, the life cycle analysis of the charging station, which considers its environmental impacts from cradle to grave, can provide a comprehensive understanding of the environmental cost.

While no specific formula is provided, the environmental charging station cost can be declared as a combination of factors such as carbon emissions, air pollutants, resource depletion, and the ecological impact associated with the charging infrastructure’s life cycle. The quantification of these factors can be formulated through environmental impact assessments and life cycle assessments to determine the overall environmental cost of the charging station.

6 $${F}_{3}={e}_{{\text{CO}}2}{\sum }_{j\epsilon {J}_{CS}}{\sum }_{i\epsilon {J}_{{CN}_{i}}}{C}_{j}{pn}_{i}{\sigma }_{ij}{d}_{ij},$$

7 $${e}_{{\text{CO}}2}=\frac{{e}_{elec}{E}_{1 \, {\text{km}}}}{{\eta }_{chrg}},$$

where e_co2_ carbon emission, ${{\text{e}}}_{{\text{elec}}}$ greenhouse gas (GHG) factor, ${{\text{E}}}_{1\mathrm{ km}}$ is the power consumption by a car running 1 km, ${\mathrm{and \eta }}_{{\text{chrg}}}$: is the charging efficiency.

### Road/user considerations

#### Service radius

In the process of determining optimal locations for FCS, several factors should be taken into consideration. Two important factors are considered, as follows:

- *Meeting Daily charging requirements and minimizing range anxiety* The placement of FCS should be designed to adequately meet the daily charging needs of customers while minimizing range anxiety. This can be achieved by ensuring that FCSs are placed near each other. By having FCSs located within a reasonable distance from each other, EV owners can have confidence that they will be able to find a charging station nearby when needed, reducing concerns about running out of battery power.
- *Efficient resource utilization* It is important to save resources by preventing FCS from being too closely spaced. Excessive clustering of charging stations can lead to inefficient usage of resources, such as land, infrastructure, and electrical capacity. To address this, the concept of the Service Radius is introduced. The Service Radius represents the maximum route an EV can take with its remaining SoC to reach the nearest FCS. By incorporating the Service Radius into the planning process, a balance can be achieved between the proximity of FCS and Range Anxiety reduction. This approach ensures that EVs are within a suitable distance of a charging station, reducing range anxiety and preventing excessive resource utilization by avoiding unnecessary clustering of FCS.

By considering the daily charging needs of clients, minimizing range anxiety, and optimizing resource allocation, the placement of FCS could achieve an efficient and effective charging infrastructure. This can be accomplished by finding the right balance between the proximity of FCS and range anxiety^75^.

#### Model of FCS capacity determination

In planning FCS capacity, customer convenience is considered by adjusting the quantity of charging slots available within an acceptable spent charging time limit. Queueing theory, specifically the M1/M2/Z queue model, shows EVs charging at the station and determines the vehicle waiting time before charging.

The M1/M2/Z queue model was chosen because it effectively captures the characteristics of EVs charging at FCS. It assumes that EVs’ inter-arrival rate (λ) and service rate (µ) at the FCS are autonomous. The M1 component of the model represents a queue where EVs’ mean arrival rate (T_λ_) follows the Poisson distribution. The M2 component represents the exponential distribution of the service time with a mean (Tµ). Z represents the number of electric vehicles charging concurrently.

Using the M1/M2/Z queuing model, the capacity planning of FCS can be optimized to balance customer convenience, waiting times, and resource utilization. This optimization guarantees that the number of charging slots is adjusted to minimize waiting times for customers while maintaining efficient charging operations at the FCS. By considering customer convenience and efficient resource allocation through the application of queueing theory and the M1/M2/Z queuing model, the capacity planning of FCS could provide an optimized charging experience for EV owners^73^.

### Uncertainties associated with EVFCS planning

#### The battery capacity

The probability density functions (PDFs) indicating the EV battery capacity (Cap) for different electric vehicle types are accessible in the EV dataset. Utilized the Monte Carlo simulation MCS, illustrated in Eqs. (8) and (9). PDF parameters are specified in Eq. (8) for the Gamma distribution and Eq. (9) for the Normal distribution^76^. In the MCS, Cap values for each EV are generated based on their respective PDFs and associated constraints. If the generated capacity falls outside the specified maximum and minimum energy constraints, the process is iterated until the capacity complies with these constraints.

8 $$f\left(Cap,\alpha ,\beta \right)= \frac{1}{{\beta }^{\alpha }\Gamma (\alpha )}{Cap}^{\alpha -1}{e}^{\frac{-Cap}{\beta }},$$

9 $$f\left(Cap,\mu ,\sigma \right)= \frac{1}{\sigma \sqrt{2\pi }}{e}^{\frac{{-(Cap-\mu )}^{2}}{{2\sigma }^{2}}},$$

where ɑ and β express the shape parameter and scale parameter of the pdf of Gamma distribution, respectively; μ and σ express the mean and standard deviation of the pdf of Normal distribution.

#### The route traveled

EVs databases contain information about the maximum distance range of different EVs based on their maximum battery capacity (Ran_mc_). The relationship between Ran_mc_ and C_B_ (battery capacity) assumes that the SOC of the EV varies linearly with the actual travel distance. This assumption is supported by mathematical models described in Refs.^77,78^.

The distance range of an EV with the available capacity of the battery (${Ran}_{ac}$) is represented by Eq. (10), while the EV’s travel range considering the ${SOC}_{cs}$ is represented by Eq. (11). The value of $\eta$, used in these equations, is determined through polynomial fitting, as explained in Ref.^79^.

10 $${Ran}_{ac}=\eta \left({SOC}_{i}-{SOC}_{c}\right){Ran}_{mc}$$

11 $${Ran}_{sc}=\eta {SOC}_{c}{Ran}_{mc},$$

where η: is the coefficient of energy efficiency, which is presented to consider the energy loss of an EV resulting from the acceleration and deceleration processes.

#### The travel starting time

Determining the travel starting time (t_s_) relies on the distribution of travel starting times obtained from a statistical survey^79^. This survey provides information on the patterns and frequencies of when people typically begin their journeys. The travel starting time distribution can be derived by analyzing these data, allowing for realistic simulations of travel behaviors and patterns.

#### Geographic information

It is possible to model transportation-related uncertainties well using Origin–Destination (OD) analysis^80^. This analysis requires specific information to be collected, including the geographical data of the planning region, the estimated number of EVs, the starting time of travel, and the OD matrix that models the mobility of EVs. The OD matrix, which outlines the movement patterns between origins and destinations, is typically obtained from local transport Authorities through transport surveys or intelligent transportation systems^77,78^.

## Effects of EV adoption^53^

**Figure 8:**
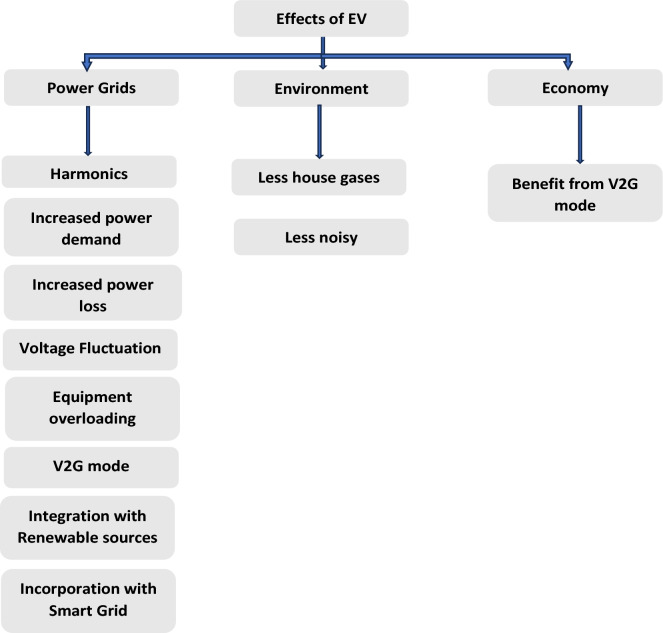
Outline of the impacts of electric vehicles ^87^.

As electric vehicles become more widespread as a response to the imperative of reducing CO_2_ emissions, the need for electrical power is escalating. Increasing electric vehicle use substantially impacts the grid’s power quality, environment, and economy, encompassing both positive and negative effects^81^. Figure 8 provides an overview of these effects, highlighting their impact on the power quality of the grid, environment, and economy. The charging methods for electric vehicles can introduce voltage fluctuations, notches, flickering, imbalances, sag, swell, and harmonics, imposing limitations on the power quality of the grid. To address these challenges, improvements in grid technology, including Vehicle-to-Grid (V2G) integration, using control system designs, smart grids, renewable energy sources, innovative technologies, and the complexity of power system networks, are necessary. However, it is important to note that these enhancements may increase the overall system cost and introduce complexity that could compromise efficiency. On a positive note, Reduced CO2 emissions and better air quality are benefits of using electric vehicles compared to conventional vehicles. Additionally, their silent operation has the potential to enhance the environment by reducing noise levels^82–86^.

## Previous FCSs studies

Proper planning of FCSs is a critical issue for the wide acceptance of EVs, but the additional load from fast charging may cause drawbacks to the grid system. These impacts include increasing power losses^88^, voltage disturbances^89^, phases unbalance^90^, equipment overloading (such as distribution lines and transformers)^84,85^, increasing power demand^91,92^, and power quality issues^93–96^.

Furthermore, uncertainties related to the location of charging demand, power and energy requirements, charging levels, charging profiles, charging patterns, and driving patterns complicate the evolution of EVs’ impact on the grid distribution system^75^. To address these issues, numerous studies have been conducted to analyze and mitigate their impacts using appropriate algorithms. Twenty-Four related studies^97–119^ will be discussed in the following subsections to define the main characteristics of recent approaches and research gaps in FCS planning.

### FCS studied characteristics

#### Cost optimization

Cost minimization is a significant concern in EV systems. This involves minimizing investment costs for charging infrastructure, optimizing operation and maintenance costs, considering different cost factors (e.g., equipment expenses, installation expenses, operational expenses, penalties), and maximizing the economic viability of these systems. The optimization process may consider social costs, emissions penalties, and the impact on power quality cost^97–99^.

#### Power quality and grid stability

A key characteristic is ensuring power quality and grid stability. This involves maintaining voltage stability, minimizing voltage deviations and power losses, managing reactive power, and addressing the effect of renewable energy integration and EV charging on grid stability and power quality. The problem formulation modeling may be complicated to include several dynamics related to FCS’s electrical behaviors, such as voltage profile, power losses, load fluctuations, harmonics distortion… etc.^98–103^.

#### Electric vehicle charging stations infrastructure

The development and optimization of EV charging infrastructure is another crucial issue. This involves determining the optimal sizing and allocation for charging stations, considering the capacity and number of stations needed, optimizing the charging schedule to minimize waiting times and maximize utilization, and addressing the drawbacks of charging on the power grid^100,102^.

#### User satisfaction and convenience

Providing a satisfactory user experience and convenient charging options for EV users are important characteristics. This includes minimizing waiting times at charging stations, optimizing charging schedules to meet user preferences, ensuring reliable and accessible charging infrastructure, and considering factors such as queuing time, service radius, and user behavior^97,104–108^.

#### Renewable energy

Some models consider the integration of photovoltaic (PV) systems^93^, wind energy^101,108^, and other renewable sources^98^ with EVs and in the distribution network. Consequently, optimization models consider multiple factors such as intermittent renewable energy generation, energy storage system management, vehicle arrival patterns, distribution network characteristics, and load uncertainties.

Uncertainties, such as solar irradiance, load required, electricity tariff, EV load characteristics, traffic situations, and charging demand patterns, are considered in certain models. The optimization models aim to find optimal solutions that balance technical, economic, and environmental benefits, ensuring efficient and sustainable operation of the EV charging infrastructure.^99,101,103,108,109^.

#### Capacitors

These can be utilized as a form of short-term storage for energy in the grid. While batteries are commonly used for energy storage in renewable energy systems and EVs, capacitors offer some unique short-term advantages. Capacitors can be used with batteries to provide additional power and energy support. They can help in regenerative braking systems, smoothing out power fluctuations, and delivering high power for rapid charging. However, for long-term energy storage, batteries are typically the preferred choice^111^.

#### Environmental impact

The environmental impact of electric vehicle systems is a concern. This involves reducing greenhouse gas emissions, minimizing reliance on fossil fuels, promoting sustainable energy practices, and assessing these systems’ overall environmental benefits and trade-offs^97,98,100,112^.

### Objective functions

The objective functions in the analyzed studies vary from profit maximization and power loss minimization to voltage stability, installation and operation cost minimization, utilization of renewable energy sources, social cost minimization, improvement in voltage profile and quality, maximization of return on investment (ROI) and net present value (NPV), improvement in reliability, and active/reactive power optimization. As explained in the following paragraphs, these objective functions reflect the diverse goals and considerations in optimizing the planning, operation, and allocation of charging infrastructure in distribution networks.

#### Profit maximization

Several publications focus on maximizing profit by considering various costs such as investment expenses, operational expenses, maintenance expenses, and penalties. The optimization frameworks proposed aim to determine optimal planning and operation strategies for charging stations while considering factors such as vehicle arrival patterns, fitful and effective management of energy storage systems, and solar photovoltaic power^107,111,113,114^.

#### Maximization of return on investment (ROI) and net present value (NPV)

Publications focus on maximizing the NPV during the project’s life cycle for optimal battery charging or swap station planning. The objective is to assess the project’s cost–benefit analysis and ensure a positive return on investment^107,115,116^.

#### Maximize utilization of renewable energy sources (RES)

Some publications focus on maximizing the usage of renewable energy sources in grid-connected EVs. The goal is to reduce EV systems’ construction expenses and energy volatility on the grid^97,108,109,117^.

#### Social cost minimization

Calculating the precise social cost of charging stations involves a complex analysis that requires considering various factors and their associated economic and social impacts. This may include factors like air pollution, climate change, public health, energy security, employment, and equity. It is worth noting that calculating the social cost is a challenging issue because of the complicated factors involved and the uncertainties associated with future developments. Different methodologies and assumptions can lead to varying results. Therefore, it is important to approach the calculation transparently and consult relevant experts or studies to ensure a comprehensive and accurate assessment. References^104,118^ propose an optimization technique to reduce the annual social cost of the EVs charging systems. The objective is to consider multiple types of charging facilities and determine optimal allocation schemes that minimize the overall social cost.

#### Power loss minimization

Some publications aim to reduce losses in grid power by optimizing the location and capacity of distributed generation units (DGs) and EV charging stations. The goal is to minimize energy losses and enhance voltage stability and profile in the network^97,98,102,104,110–112^.

#### Voltage stability and deviation

Certain publications consider voltage stability and deviation as the objective functions. The optimization frameworks aim to allocate DG modules, energy storage systems (BESS), and EV charging systems in a way that optimizes power loss, voltage stability, and voltage fluctuations in the distribution grid.

#### Installation and operation cost minimization

In some publications, the minimization of installation and operation costs is considered an objective function. The optimization models aim to specify the optimal location and sizing of DGs, EV charging systems, and other charging infrastructure while considering factors such as investment costs, maintenance costs, and additional conductors required for system reconfiguration^100,116^.

#### Voltage profile and quality improvements

Several publications aim to enhance the voltage profile and quality of the distribution grid by optimizing the sizing and location of charging stations, DGs, and other renewable sources. The objective is to minimize voltage fluctuations, improve thermal stability, and enhance the overall voltage profile^97,98,100–102,111–113,119^.

#### Improvement in reliability and reduction of energy losses

Some publications aim to minimize energy losses and enhance the reliability of the distribution grid by optimizing the location and operation of EV charging systems. The objective is to reduce active power losses and the energy not supplied to the network while ensuring a reliable power supply^110,111^.

#### Addressing specific factors

The objective function considers additional factors, such as flood hazards, demand response, user satisfaction, and integration with distributed generations (DGs). These factors contribute to optimizing the charging stations’ location, size, and operational strategy^105,119^.

It is essential to note that the specific weights and priorities assigned to each objective can differ depending on the specified context, stakeholder preferences, and local requirements. The objective function may also be refined and adjusted based on additional considerations and constraints specific to the charging station planning process.

#### Vehicle to grid V2G

V2G refers to a system where EVs can discharge power from their batteries back to the grid, providing grid support and potentially earning revenue for vehicles or station owners. V2G can provide grid operators with additional flexibility by utilizing the battery energy storage of EVs during periods of peak demand or grid instability. It can help balance the grid by injecting power during peak demand periods or when intermittent renewable generation is low.

V2G has the potential to decrease peak electricity demand by enabling EVs to supply power during periods of high load. This significantly reduces the load on the power system and minimizes the necessity for extra generation capacity installation. Consequently, this can result in cost savings and enhanced grid reliability. While numerous studies have explored the advantages and limitations of V2G, only a limited number have examined it solely as an operational mode to assess the behavior of EV-planned charging stations^97,104^.

Peak electricity demand could decrease due to V2G technology, improve grid reliability, and provide cost savings. While research on the benefits and limitations of V2G exists, more attention should be given to its operational mode in evaluating EVs’ planned charging stations. Additionally, incorporating V2G into power system critical analyses can optimize these assessments. However, this aspect is often overlooked in FCS planning research.

### Optimization techniques

Different optimization algorithms such as mixed integer non-linear problem (MINLP)^97,98,102,106^, Particle Swarm Optimization (PSO)^97,104,105,107,111^, Genetic Algorithm (GA)^112^, Non-dominated Sorting Genetic Algorithm II (NSGA-II), Differential Evolution (DE)^97,102,119^, Grey Wolf Optimization (GWO)^109^, and Immune Algorithm^105^ are used to solve the optimization issues.

Most optimization techniques proposed for addressing nonlinear and uncertain problems are well-known metaheuristic optimization methods. These techniques maintain a population of candidate solutions, such as individuals or particles, and systematically enhance them through iterations. By repeatedly updating the candidate solutions across multiple generations or iterations, they aim to converge toward optimal or near-optimal solutions. Additionally, these methods introduce randomization into their search process, enabling them to break free from local optima and explore improved solutions. Moreover, Hybrid Optimization of Multiple Energy Resources software HOMER is proposed in many studies to optimize both technical and economic FCS behaviors^98,117^. The main features of previous studies are summarized in Table 3.

**Table 3:** Main features of previous studies.

Pub.	Considerations	Optimization techniques	Objective function
118 (2021)	Arrival pattern of vehicles Irregular solar photovoltaic power Management of energy storage systems The costs of investments, operations, upkeep, and penalties	AMPL with a Gurobi solver	Profit maximization
115 (2022)	Electric vehicle charging stations (EVCS) and distributed generators (DG) G2V and V2G operation modes	PSO, or simultaneous particle swarm optimization	Minimizing power losses
104 (2022)	Distribution network facilities for DG units, battery energy storage systems (BESS) units, and EVCS facilities Uncertainties related to load usage, solar irradiance, and power costs	Reinforcement learning (RL)-based algorithm Multi-stage, hybrid optimization scheme	Minimization of power losses Voltage fluctuations Operation and maintenance cost CO2 emission cost
121 (2021)	The optimum possible configuration of the imbalanced redial distribution scheme for the EVCS	Particle Swarm Optimization technique (PSO)	Reducing costs associated with installing and erecting tie-lines
122 (2021)	Taking EV load uncertainty, grid-connected EVCS with renewable energy sources (RES) planning is investigated	HOMER software	Maximizing RES utilization. Minimizing the construction cost The grid’s fluctuating electricity supply
112 (2021)	The grid’s and electric vehicle owners’ connected benefit	Voronoi diagram combined with improved PSO	Minimizing the social cost
117 (2013)	Environmental issues and service radius of EVCS	Modified primal–dual interior point algorithm	Minimizing grid loss
123 (2018)	A combination of multi-type charging facilities	Mixed integer second-order cone programming	Minimizing the social cost of the whole charging system
105 (2017)	Optimal placement/sizing of EVCS in the city of Allahabad	Hybrid algorithm based on genetic algorithm and PSO	Reducing the cost of developing, constructing, and maintaining
106 (2015)	Grid voltage profile The day-ahead load characteristic’s hourly peak demand ratio, wind speed, and sun irradiation	A differential evolution algorithm	Minimizing power losses Charging costs of EVs
120 (2014)	Stations for fast battery swapping and charging in distribution networks	A modified differential evolution algorithm	Maximization of net present value
112 (2018)	The location and sizing of FCSs and distributed generations (DGs), considering constraints like the proportion of EVs in each zone and the maximum number of FCSs possible given the planned system’s road and electrical infrastructure	A mixed integer non-linear problem (MINLP) and Non-dominated sorting genetic algorithm II (NSGA-II)	Minimize of the investment cost of CSs and DG units voltage deviation, and power losses
110 (2020)	The integration of natural gas, transportation, and active distribution networks Strategies of renewable energy generation and the effect of traffic flow	A mixed-integer nonlinear program problem	Environmental performance
114 (2021)	Uncertainty in PV, wind, and load power	Bi-level met heuristic-based solution The grey wolf optimization (GWO)	Maximizing RES and EV decision variables
107 (2020)	Static, dynamic, and heuristic on balanced radial distribution system	Multiple objective particle swarm optimization (MOPSO)	Minimizing losses Reliability of the system thermal stability
116 (2021)	Reactive power compensation effect	A hybrid of grey wolf optimization and PSO	Minimizing the active power loss Maximizing the net profit
111 (2022)	The user’s satisfaction and the ease of charging are considered	An immune algorithm	Maximum electric vehicle user satisfaction
124 (2022)	Three types of flooding are considered: riverine flooding, sea-level rise-induced chronic flooding, and coastal flooding brought on by hurricanes	Non-dominated sorting genetic algorithm-III (NSGA-III) and the technique for order of preference by similarity to ideal solution (TOPSIS)	Decrease the charging convenience Decrease the impact of flood hazards
119 (2022)	A planning strategy for PV/storage fast charging stations that considers demand response	Non-dominated sorting genetic algorithm (NSGA-II)	Maximizing the revenue from fees paid to service providers Minimizing the total charging time of EVs
113 (2020)	Both traffic and the power distribution grid, the constraints of safe and stable grid operation	A linearized generalized Bender’s decomposition algorithm	Maximizing the profit
102 (2021)	Convenience for EV users, station economic profit, the effect on distribution systems, and the impact on the environment	Multi-objective binary and non-dominated sorting genetic algorithm	Minimizing queuing time, station economy
103 (2023)	A hybrid nuclear-renewable energy system Emission COE, GHG emissions, O&M cost, and ROI	HOMERPro software	Minimizing the cost of different emissions, O&M, and ROI

## Research challenges

### Operational dynamics of V2G stations

A substantial research lacuna persists in delineating the operational dynamics of Vehicle-to-Grid (V2G) technology. The behavior and functionality of planned V2G stations demand in-depth scrutiny to comprehensively comprehend their nuanced operational mode.

### Underemphasis on V2G studies

Predominant research efforts in fast charging station planning predominantly revolve around losses and voltage stability, sidelining pivotal studies related to V2G technology. A critical challenge lies in redirecting focus towards a more inclusive examination of the operational intricacies inherent in V2G stations.

### Holistic approach to EV infrastructure challenges

While tackling challenges associated with fast-charging infrastructure, there exists a demand for a more holistic perspective. This entails addressing technical intricacies and comprehending the economic and environmental ramifications, ensuring a well-rounded comprehension of challenges associated with the widespread adoption of electric vehicles (EVs).

### Optimization and standardization of renewable energy integration

Despite the recognized advantages of incorporating renewable energy sources and energy storage systems into fast charging networks, research endeavors should optimize and standardize these integration methodologies. This necessitates addressing challenges related to intermittent scalability and economic feasibility.

### Integrated planning approaches

The current suite of planning approaches, while informative, requires augmentation to holistically address challenges about optimizing charging station locations, capacity planning, and grid integration concurrently. Achieving equilibrium in these considerations poses a multifaceted challenge demanding an integrated and systematic planning paradigm.

### Dynamic forecasting for EV adoption

The dynamic landscape of electric vehicle adoption introduces challenges in forecasting and planning for the escalating demand for fast-charging infrastructure. Research initiatives should pivot towards developing adaptive models and strategies capable of accommodating the evolving dynamics of EV adoption and user behaviors.

### Economic viability metrics for charging infrastructure

Ensuring the economic viability and sustained functionality of charging infrastructure remains a formidable challenge, particularly in regions marked by fluctuating energy costs and evolving market dynamics. Research endeavors should prioritize the development of models that intricately consider economic factors to underpin the enduring success of fast charging stations.

The elucidation and resolution of these research challenges are paramount for propelling the advancement and sustainability of electric vehicle infrastructure, thus facilitating a seamless transition towards an electric mobility-dominated future.

## Conclusion

As the penetration of electric vehicles (EVs) continues to surge in the vehicle market, presenting a viable solution to environmental concerns and reducing reliance on fossil fuels, establishing an efficient and reliable fast-charging infrastructure becomes paramount. This review thoroughly examines the planning strategies and considerations integral to deploying electric vehicle fast charging stations.

The paper underscores the imperative for fast charging infrastructure as the demand for EVs escalates rapidly, highlighting its pivotal role in facilitating the widespread adoption of EVs. The review acknowledges and addresses the challenges associated with planning for such infrastructure.

A key focal point of this review is exploring the benefits of integrating renewable energy sources and energy storage systems into networks with fast charging stations. By leveraging clean energy and implementing energy storage solutions, the environmental impact of EV charging can be minimized, concurrently enhancing sustainability.

Moreover, the review delves into existing planning approaches, simulation models, and optimization techniques for designing and operating fast-charging networks. These methodologies offer valuable insights into optimizing charging station locations, capacity planning, and grid integration, ensuring efficient resource utilization and maximizing overall infrastructure effectiveness.

The potential of Vehicle-to-Grid (V2G) technology emerges as a notable aspect explored in this review, showcasing its ability to address peak electricity demand by utilizing EVs to supply power during periods of high load. This approach alleviates strain on the power grid, reducing the necessity for additional generation capacity, leading to cost savings, and enhancing grid reliability. Despite the extensive exploration of V2G advantages and limitations in existing studies, only some have specifically assessed its operational mode to evaluate the behavior of planned stations.

Furthermore, properly planning and integrating V2G as a valuable temporary additional power source can optimize critical analyses of power systems, including contingency and restoration assessments. However, it is noteworthy that existing research on fast charging station planning predominantly focuses on losses and voltage stability, often overlooking these critical V2G studies.

## Data Availability

The datasets used and generated during the current study are available from the corresponding author upon reasonable request.
